# High Expression of Ecto-Nucleotidases CD39 and CD73 in Human Endometrial Tumors

**DOI:** 10.1155/2014/509027

**Published:** 2014-02-24

**Authors:** Elisabet Aliagas, August Vidal, Laura Texidó, Jordi Ponce, Enric Condom, Mireia Martín-Satué

**Affiliations:** ^1^Departament de Patologia i Terapèutica Experimental, Facultat de Medicina, Campus de Bellvitge, Universitat de Barcelona, Pavelló de Govern, 4a Planta, Lab. 4145, C/Feixa Llarga s/n, 08907 L'Hospitalet de Llobregat, Barcelona, Spain; ^2^Institut d'Investigació Biomèdica de Bellvitge (IDIBELL), Barcelona, Spain; ^3^Servei d'Anatomia Patològica, Hospital de Bellvitge, Barcelona, Spain; ^4^Servei de Ginecologia, Hospital de Bellvitge, Barcelona, Spain

## Abstract

One of the strategies used by tumors to evade immunosurveillance is the accumulation of extracellular adenosine, which has immunosupressive and tumor promoting effects. The study of the mechanisms leading to adenosine formation at the tumor interstitium are therefore of great interest in oncology. The dominant pathway generating extracellular adenosine in tumors is the dephosphorylation of ATP by ecto-nucleotidases. Two of these enzymes acting sequentially, CD39 and CD73, efficiently hydrolyze extracellular ATP to adenosine. They have been found to play a crucial role in a variety of tumors, but there were no data concerning endometrial cancer, the most frequent of the invasive tumors of the female genital tract. The aim of the present work is to study the expression of CD39 and CD73 in human endometrial cancer. We have analyzed protein and gene expression, as well as enzyme activity, in type I endometrioid adenocarcinomas and type II serous adenocarcinomas and their nonpathological endometrial counterparts. High levels of both enzymes were found in tumor samples, with significantly increased expression of CD39 in type II serous tumors, which also coincided with the higher tumor grade. Our results reinforce the involvement of the adenosinergic system in cancer, emphasizing the relevance of ecto-nucleotidases as emerging therapeutic targets in oncology.

## 1. Introduction

Extracellular adenosine concentration increases under metabolically stressful conditions, notably in the tumor microenvironment [1], where hypoxia is frequently given [2, 3]. Such accumulation of adenosine mediates, through four distinct receptors (A1, A2A, A2B, and A3), complex and diverse effects that lead to tumor immunoescape [4]. This includes cytoprotection and growth promotion of tumor cells [5, 6], angiogenesis increase [7, 8], and suppression of effector (antitumor) T cells [9].

Although cells are provided with adenosine transporters, the main source of this nucleoside in the tumor interstitium is the hydrolysis of extracellular ATP, which also accumulates in tumors, by membrane enzymes known as ecto-nucleotidases [6, 10]. Different families of these enzymes, acting extracellularly, are responsible for the generation of adenosine from adenine nucleotides (i.e., ATP, ADP, or AMP): (1) the ectonucleoside triphosphate diphosphohydrolase (E-NTPDase) family, that includes four plasma membrane-bound members: NTPDase1 (CD39), NTPDase2, NTPDase3, and NTPDase8; these enzymes are differentially expressed and hydrolyze with different affinities nucleoside triphosphates and diphosphates to their monophosphate derivatives (e.g., ATP and ADP to AMP); (2) the ectonucleotide pyrophosphatase/phosphodiesterase (E-NPP) family, capable of hydrolyzing nucleoside triphosphates to monophosphates and pyrophosphate (PPi), such as ATP to AMP and PPi; (3) the alkaline phosphatase (AP) family, that includes ubiquitous enzymes degrading broad range of substrates, such as adenine nucleotides and PPi, releasing inorganic phosphate (Pi); (4) the 5′-nucleotidase family, with only one member attached to the outer plasma membrane, the ecto-5′-nucleotidase (CD73), a glycosyl phosphatidylinositol-linked membrane-bound glycoprotein that efficiently hydrolyses AMP to adenosine [11–13].

Two members of ecto-nucleotidases families, the E-NTPDase CD39 and the 5′-nucleotidase CD73, acting sequentially, seem to have a crucial role in tumor-immune cell interaction [6]. They both are expressed not only by infiltrating immune cells but also by tumor cells, and their expression is regulated by hypoxia [10, 14]. Increased CD39 and CD73 expression has been described in various cancer types, mostly in correlation with a poor prognosis [15–17]. Both molecules are considered promising therapeutic targets in oncology, and CD73 has already been proven to inhibit tumor growth and metastasis in a breast cancer model in mice [18–20]. However, until now there were not available data concerning ecto-nucleotidases expression in endometrial cancer (EC). EC is the most frequent of the invasive tumors of the female genital tract. There are two clinicopathological variants: the estrogen-related, type I, endometrioid carcinoma, and the nonestrogen-related, type II, nonendometrioid carcinoma [21]. Although there are different molecular alterations that have been already identified in EC, with different prevalence between tumors [22, 23], there is need to decipher the complete molecular profile of EC pathogenesis to improve diagnosis and favor the design of new therapeutic strategies.

The aim of the present work was to study the expression of CD39 and CD73 in endometrioid (type I) and serous (type II) EC when compared with nontumoral endometrium. To achieve this objective, protein and gene expression experiments, as well as *in situ *enzyme activity assays, were performed on human endometrial adenocarcinoma samples and their nontumoral endometrial counterparts.

## 2. Materials and Methods 

### 2.1. Samples

The ethical principles of this study adhere to the Declaration of Helsinki, and all the procedures were approved by the ethics committee for clinical investigation of Bellvitge Hospital. Endometrial samples from adenocarcinoma (endometrioid and serous types) and their corresponding nontumoral tissue (if present) were obtained from hysterectomy specimens at the Service of Gynecology of Bellvitge Hospital. Fresh samples were cut, embedded in O.C.T. freezing media (Tissue-Tek; Sakura Finetek, Zoeterwoude, The Netherlands), snap-frozen in a Shandon Histobath 2 (Neslab Instruments Inc., USA) at the Service of Pathology, and stored at −80°C until used. Alternatively, endometrial samples were obtained from the Tumor Bank of Bellvitge Biomedical Research Institute (IDIBELL).

Fifteen endometrioid adenocarcinomas (13 grade 1, 2 grade 2; all FIGO stage I) (56–82 years old, median 61) and fourteen serous adenocarcinomas (grade 3; 9 FIGO stage I, 2 FIGO stage II, and 3 FIGO stage IV) (63–86 years old, median 77), and their adjacent nontumoral endometrium were used in this study.

### 2.2. Endometrial Touch Prep Technique

Touch preparations of endometrial cancer tissue samples were obtained by lightly pressing the freshly cut tumor surface on clean glass microscope slides, thus generating a tumor cell imprint. Imprints were immediately air-dried and stored at −20°C until further processing. Six endometrioid adenocarcinoma tissue samples were used to generate the touch preparations.

### 2.3. Immunolabeling Experiments

Immunohistochemistry and immunofluorescence experiments were performed as previously described for human endometrial samples [24]. Briefly, tissue sections of 10 *μ*m thick and touch preparations were fixed in 10% phosphate-buffered formalin mixed with cold acetone (Merck, Darmstadt, Germany) for 2.5 minutes. Fixed samples were rinsed with PBS and preincubated for 1 hour at room temperature (RT) with PBS containing 20% normal goat serum (Gibco, Paisley, UK) and 0.2% gelatin (Merck). Samples were then incubated overnight at 4°C with the following primary antibodies: anti-human CD39 clone BU61 (Ancell Corporation, Minnesota, MN, USA) at 1/500, mouse monoclonal anti-human ecto-5′-nucleotidase (CD73) clone 4G4 (Abcam, Cambridge, UK) at 1/50, and rabbit monoclonal anti-human cytokeratin 19 (CK19) clone EPR1579Y (Abcam) at 1/200. After three washes in PBS, samples were incubated for 1 hour at RT with the appropriate secondary antibodies: horseradish peroxidase-conjugated goat anti-mouse (EnVision + system; DAKO, Carpinteria, USA), Alexa Fluor 488- or 555-goat anti-mouse or anti-rabbit (Life Technologies, Paisley, UK). Secondary antibody alone was routinely included as control for the experiments. Nuclei were counterstained with haematoxylin or, alternatively, in fluorescence assays, To-Pro-3 or DAPI (Life Technologies) were used to visualize the nuclei. Samples were mounted with Fluoromount aqueous mounting medium (Sigma-Aldrich, Sant Louis, Missouri, MO, USA). The results were observed and photographed under a light Leica DMD 108 microscope (Leica Microsystems, Wetzlar, Germany) or, in fluorescence assays, under a Nikon Eclipse E-800 microscope (Nikon, Tokyo, Japan) or under a Leica TCS-SL spectral confocal microscope (Leica).

Results from both tissue samples and touch preparations were independently evaluated by at least two observers. Staining distribution was recorded. Label intensity was scored as negative (−), intermediate (+), or strongly positive (++).

### 2.4. *In Situ* Enzyme Activity Experiments

For enzyme histochemistry, ADPase and ecto-5′-nucleotidase (AMPase) activities were localized by using the Wachstein/Meisel lead phosphate method [24, 25] in tissue samples and in touch preparations. Briefly, fixed samples were preincubated for 1 hour at RT in 50 mM Tris-maleate buffer, pH 7.4 containing 2 mM CaCl_2_ and 0.25 M sucrose. Enzyme reaction was carried out for 1 hour at 37°C in the same buffer supplemented with 5 mM MnCl_2_, 2 mM Pb(NO_3_)_2_, 3% Dextran T250, and 2.5 mM levamisole, as inhibitor of alkaline phosphatases, and in the presence of 200 *μ*M ADP or 1 mM AMP, as substrate. For CD39 and CD73 inhibition experiments, 1 mM NF279 (Tocris Bioscience, Bristol, United Kingdom) and 1 mM *α*, *β*-meADP (Sigma-Aldrich) were added, respectively, to both preincubation and enzyme reaction buffers. Control assays were performed in the absence of nucleotide. The reaction was revealed by incubating with 1% (NH_4_)_2_S v/v for exactly 1 minute. Samples were counterstained with haematoxylin, mounted with Fluoromount aqueous mounting medium (Sigma-Aldrich), and observed and photographed as described above.

### 2.5. Isolation of Membrane Enriched Fraction from Tissue Homogenates

50–100 *μ*g of human tumor (endometrioid and serous endometrial adenocarcinoma) and nontumoral tissue samples were homogenized in a buffer containing 20 mM Hepes, 250 mM sucrose, 0.3 mM PMSF, 1 mM DTT, 1 mM EGTA, and 1 mM MgCl_2_ (pH 7.4) using a glass homogenizer (VidraFoc, Barcelona, Spain). After homogenization, samples were centrifuged at 600 ×g for 10 minutes at 4°C in a Beckman JA-20 centrifuge. The pellet was discarded and supernatants were centrifuged at 48,000 ×g for 20 minutes at 4°C in a Beckman TI-70 centrifuge. The resulting pellets were resuspended in a buffer containing 20 mM Hepes, 0.3 mM PMSF, and 1 mM DTT (pH 7.4). Protein concentration was determined by the method of Lowry et al. [26] using bovine serum albumin as a standard. Samples were kept at −80°C until use.

### 2.6. Enzyme Activity Assays in Plasma Membrane Enriched Tissue Homogenates

ADPase and AMPase activities were determined by measuring the amount of Pi using the malachite green colorimetric assay, as previously described [27].

### 2.7. Quantitative Real-Time PCR

Total RNA from endometrial tumor tissue samples was isolated using the RNeasy Plus Mini Kit (Qiagen, Hilden, Germany), following the manufacturer's protocol. Total isolated RNA (2 *μ*g) was reversely transcribed into complementary DNA (cDNA) using the First Strand cDNA Synthesis Kit (Fermentas, Thermo Scientific, Chicago, IL, USA).

Quantitative real-time PCR (qRT-PCR) was performed to examine the expression of *CD39*, *NTPDase2, *and *CD73 *genes. Designed large-scale TaqMan low-density array (TLDA) microfluidic cards (Applied Biosystems, Foster City, CA, USA) were used. The 384 wells of each card were preloaded with predesigned fluorogenic TaqMan probes and primers for *CD39*, *NTPDase2, *and *CD73*. cDNA (1 *μ*g) combined with TaqMan 2X Universal PCR Master Mix (Applied Biosystems) were loaded into each sample-loading port. qRT-PCR reactions were carried out using the ABI PRISM 7900HT Real-Time PCR System (Applied Biosystems). Data were collected using the SDS v2.1 software (Applied Biosystems) and analyzed by the comparative Ct (ΔΔCt) quantification method using the Expression Suite v1.0 software (Applied Biosystems). The relative expression levels of *CD39*, *NTPDase2*, and *CD73 *genes were determined using 18S mRNA as an endogenous control for normalization. Results are expressed as the mean of the relative quantification (RQ) of the tested transcripts (*n* = 7 serous adenocarcinoma samples; *n* = 7 endometrioid adenocarcinoma samples) ± the standard error of the mean (SEM). Results were obtained from five independent experiments performed using 1 *μ*g of cDNA, all with duplicate measurements. No signal was detected in nontemplate controls.

### 2.8. Statistical Analysis

Statistical analysis was performed using SigmaStat 3.2 software (SPSS Inc., Chicago, IL, USA). Values are reported as the mean ± S.E.M. Student's *t*-test was used to compare the means of two independent groups of normally distributed data.

## 3. Results and Discussion

Extracellular adenosine in tumors, mainly generated by the sequential action of ecto-nucleotidases, has immunosuppressive effects through a broad range of actions, including inhibition of antitumor T-cell function, modification of local interleukin levels, and inhibition of phagocytosis (reviewed in [28]). In this section we show and discuss our results on the expression of the ecto-nucleotidases CD39 and CD73 in type I and type II endometrial carcinomas.

**Figure 1:**
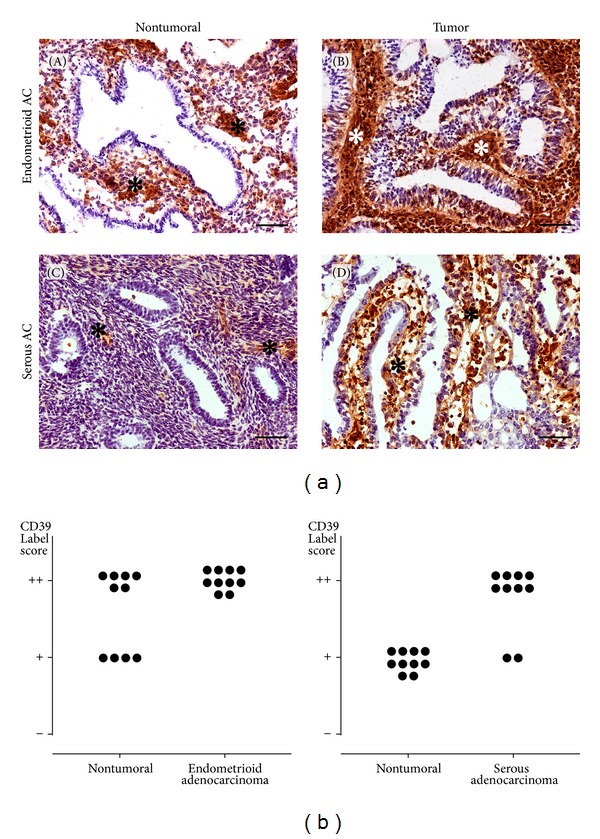
(a) Immunolocalization of NTPDase1/CD39 in nontumoral human endometria ((A), (C)) and in endometrioid (B) and serous (D) adenocarcinomas. CD39 was immunodetected in the stroma of all samples (asterisks). The expression of CD39 is remarkably higher in tumors when compared with nontumoral endometria. Scale bars = 100 *μ*m. (b) Label intensity score of CD39 in the stroma of nontumoral human endometrium compared to endometrioid (left plot) and serous (right plot) adenocarcinoma samples. Note that both types of tumors have a higher label score than their corresponding nontumoral endometria.

CD39 was immunolocalized in the stroma of both nontumoral and tumoral endometria (Figure 1(a)). For the nonpathological endometrium the expression of CD39 has already been previously described in association with stromal cells and blood vessels [24]. Here we show that label score was significantly higher for both endometrioid and serous types of tumors when compared with the corresponding nontumoral coexisting endometrium (Figure 1(b)). No CD39 labeling was found in endometrial adenocarcinoma epithelia, as demonstrated with the double staining performed with the anti-CD39 and anti-CK19 antibodies (see Supplementary Figure 1 in Supplementary Material available online at http://dx.doi.org/10.1155/2014/509027).

**Figure 2:**
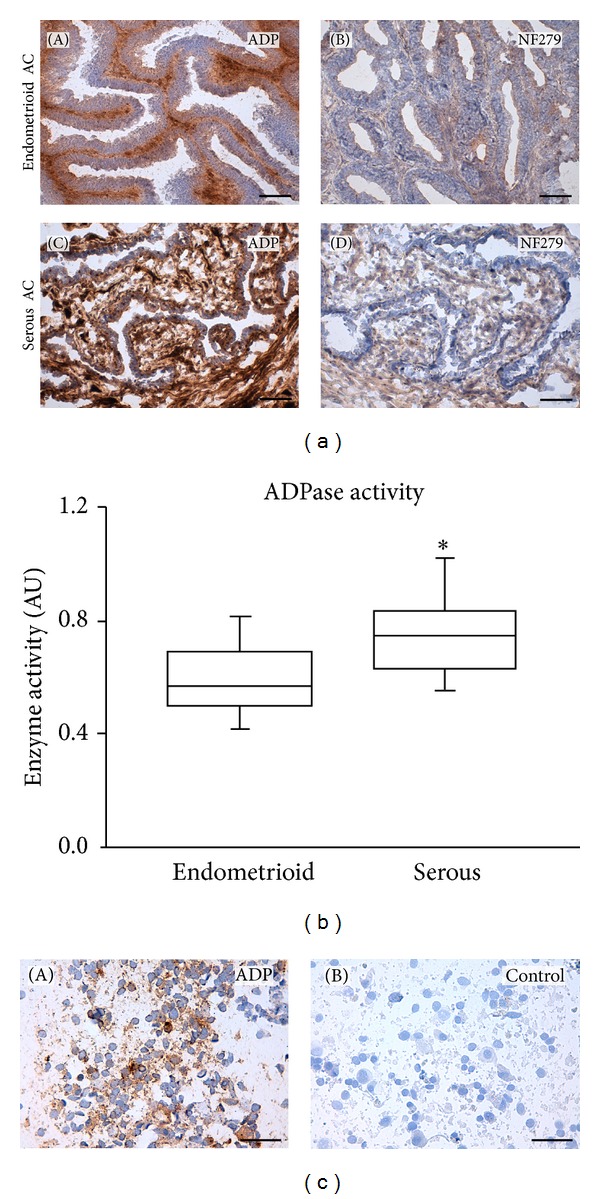
(a) Enzyme *in situ *histochemistry in endometrioid ((A), (B)) and in serous ((C), (D)) endometrial adenocarcinomas in the presence of ADP as substrate, alone ((A), (C)) or together with the inhibitor NF279 ((B), (D)). Strong ADPase activity was detected in the stroma of both types of tumors ((A), (C) dark brown deposits), while the activity was drastically reduced in the presence of the inhibitor ((B), (D)). Scale bars = 100 *μ*m. (b) ADPase enzyme activity in tissue homogenates of endometrioid (*n* = 12) and serous (*n* = 14) adenocarcinomas. Experiments were performed in triplicate for each sample. Data are represented in arbitrary units (AU). *Significant differences at *P* < 0.05. (c) ADPase *in situ *activity in endometrial touch preparations from human endometrioid adenocarcinomas using ADP as substrate (A) and in the absence of substrate (B). Scale bars = 50 *μ*m.

Strong *in situ *ADPase activity was detected in the tumor stroma, coinciding with the CD39 immunolocalization (Figure 2(a)). This activity was drastically reduced with the NTPDase inhibitor NF279, demonstrating its specificity. Equivalent results were obtained using ATP as substrate for the *in situ *activity assay (not shown). ADPase activity measured in tumor tissue homogenates demonstrated that serous (grade 3) adenocarcinomas had significantly higher activity than endometrioid (grade 1) adenocarcinomas (Figure 2(b)).

**Figure 3:**
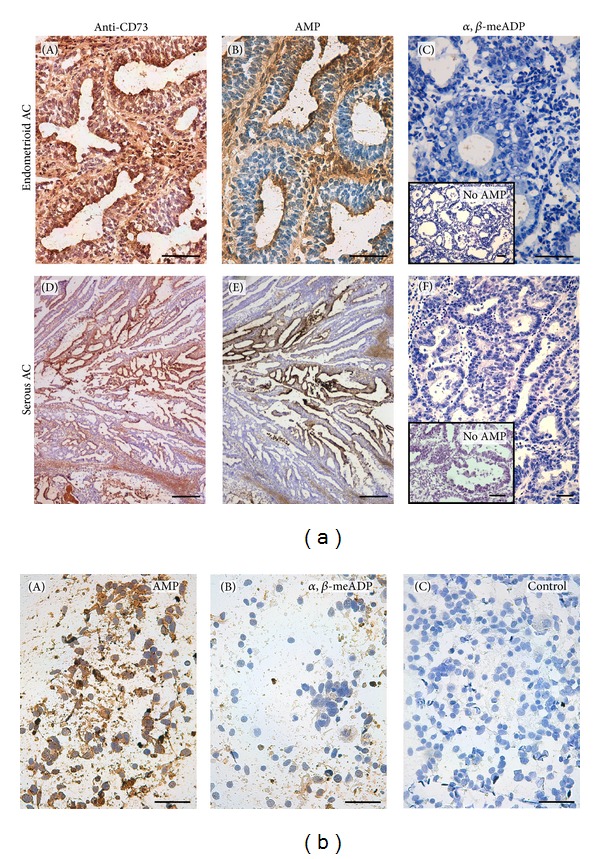
(a) Immunolocalization of ecto-5′-nucleotidase/CD73 ((A), (D)) and AMPase *in situ *histochemistry ((B), (C), (E), and (F)) in endometrioid ((A)–(C)) and serous ((D)–(F)) endometrial adenocarcinomas. CD73 was immunodetected in glandular epithelial cells and in the stroma of both types of adenocarcinomas ((A), (D)). The AMPase activity mirrors the immunolocalization ((B), (E)). The activity was abolished in the presence of *α*, *β*-meADP ((C), (F)). Insets correspond to control experiments performed in the absence of nucleotide (no AMP). Scale bars = 50 *μ*m ((A)–(C), (F), insets) and 250 *μ*m ((D), (E)). (b) AMPase *in situ *activity by enzyme histochemistry in endometrial touch preparations. Dark precipitates in (A) correspond to the AMPase activity in the presence of AMP as substrate. The inhibitor *α*, *β*-meADP completely abolished this activity (B). Control experiments were performed in the absence of substrate (C). Scale bars = 50 *μ*m.

CD73 expression was strongly immunodetected in both types of tumors, in epithelial structures and in the stroma (Figure 3(a)), thus partially colocalizing with CK19 (Supplementary Figure 2). Specific CD73 activity, demonstrated with the inhibitor *α*, *β*-meADP, matched the immunolabeled structures (Figure 3(a)). We have already previously demonstrated that the expression and activity of CD73 are abundant in nonpathological endometrium [24, 25]. Consequently, due to the high expression of CD73 in tumoral and nontumoral endometria, label score comparisons were not possible. Moreover, no differences among tumors were observed with the enzyme assays either in tissue slices or in tissue homogenates (not shown).

Immunolabeling and *in situ *enzyme activity results obtained with the touch prep technique were equivalent to those obtained with tissue slices (Figures 2(c) and 3(b)). The usefulness of the touch prep technique for diagnosis has already been demonstrated in breast cancer with 100% sensitivity and specificity in the evaluation of tumor margins at the time of the surgery [29]. This technique has also been previously used to demonstrate by immunolabeling a decreased expression of P2X7 ATP receptor in endometrial cancer cells [30] and also, recently, the relationship between p53 expression and the tumor grade [31]. However, to our knowledge, this is the first report validating the use of touch preps for enzyme activity studies, therefore opening the possibility of performing such studies in cytological samples.

**Figure 4:**
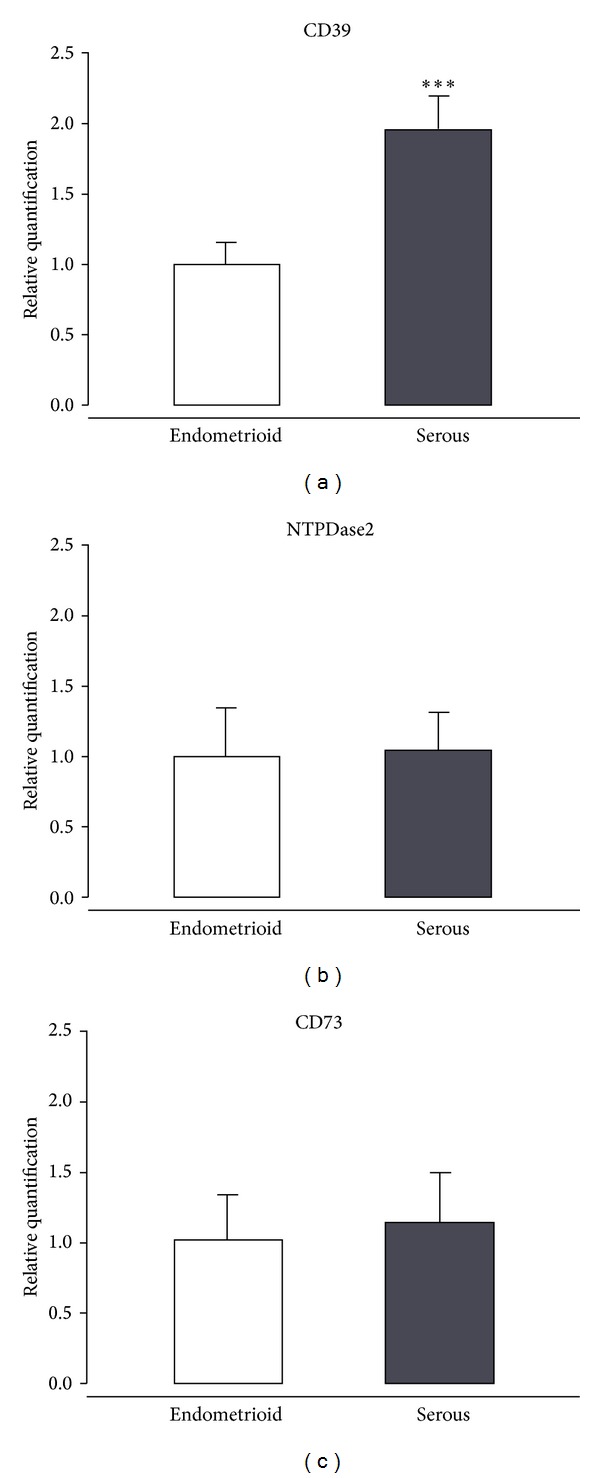
*CD39*, *NTPDase2, *and *CD73 *gene expression in human endometrial cancer tissues. Relative mRNA levels of *CD39 *(a), *NTPDase2 *(b), and *CD73 *(c) analyzed in serous adenocarcinomas (*n* = 7), normalized to *18S *mRNA levels, and expressed as fold change over endometrioid adenocarcinomas (*n* = 7). Data are expressed as mean ± SEM. ***Significantly different from endometrioid (*P* < 0.001).

In order to determine if the differences in CD39 protein and ADPase activity between tumor types also involved gene expression changes, quantitative real-time PCR analyses were performed (Figure 4). *CD39 *gene expression was 2-fold higher in serous endometrial adenocarcinoma than in endometrioid, coinciding with the data obtained with the protein (Figure 4(a)). These gene expression changes did not apply to *NTPDase2 *(Figure 4(b)), indicating that *CD39 *upregulation is not a general feature of other members of NTPDase family. No changes were detected in *CD73 *gene expression between the two tumor types (Figure 4(c)).

These results on endometrial tumors add to the list of human cancers in which CD39 is overexpressed and support the growing body of evidence that CD39 is a potential therapeutic target for cancer immunotherapy [18]. Our results also reinforce the relevance of CD73 in tumors. Antibody-based therapy and pharmacological approaches against CD73 have been reported to significantly inhibit tumor growth and improve antitumor immunity in mouse models [28, 32].

## 4. Conclusions

Endometrial adenocarcinoma tumors have significantly higher CD39 expression and activity than their nontumoral counterparts. Moreover, stronger activities are associated with type II serous tumors. This also coincides with the higher grade of these tumors, but further studies are needed to establish statistical correlations with the tumor grade in the case of type I endometrioid tumors. The consequences of this high CD39 activity in endometrial tumors are increased levels of AMP, the substrate for CD73, and also highly expressed in these tumors, which will, in turn, generate increased immunosuppressive levels of extracellular adenosine.

## Supplementary Material

The supplementary figures demonstrate that CD39 does not colocalize with CK19 (Figure 1) whereas CD73 partially colocalizes (Figure 2).
