# Supplementary material for: High Expression of Ecto-Nucleotidases CD39 and CD73 in Human Endometrial Tumors
Source: Mediators Inflamm. 2014 Feb 24;2014:509027. doi: 10.1155/2014/509027 (PMC3953595; doi:10.1155/2014/509027)

Supplementary figure legends

Supplementary figure 1. Immunolocalization of NTPDase1/CD39 and cytokeratin 19 (CK19) in human endometrioid adenocarcinomas (A-C) and endometrial touch preparations (D-F). CD39 (red) was localized in the stroma (A, D) and CK19 (green) in the epithelial cells (B, E). Merge images show that there is no colocalization between CD39 and CK19 (C, F). Scale bars = 25 µm (A-C) and 50 µm (D-F).

Supplementary figure 2. Immunolocalization of ecto-5'-nucleotidase/CD73 and cytokeratin 19 (CK19) in human endometrioid adenocarcinomas (A-C) and endometrial touch preparations (D-F). CD73 (red) was mainly localized in the apical domain of glands (A, D) whereas CK19 (green) labelled completely the glandular epithelium (B, E). Merge images show, in yellow, colocalization between CD73 and CK19 (C, F). Scale bars = 20 µm (A-C) and 40 µm (D-F).

Supplementary figure 1

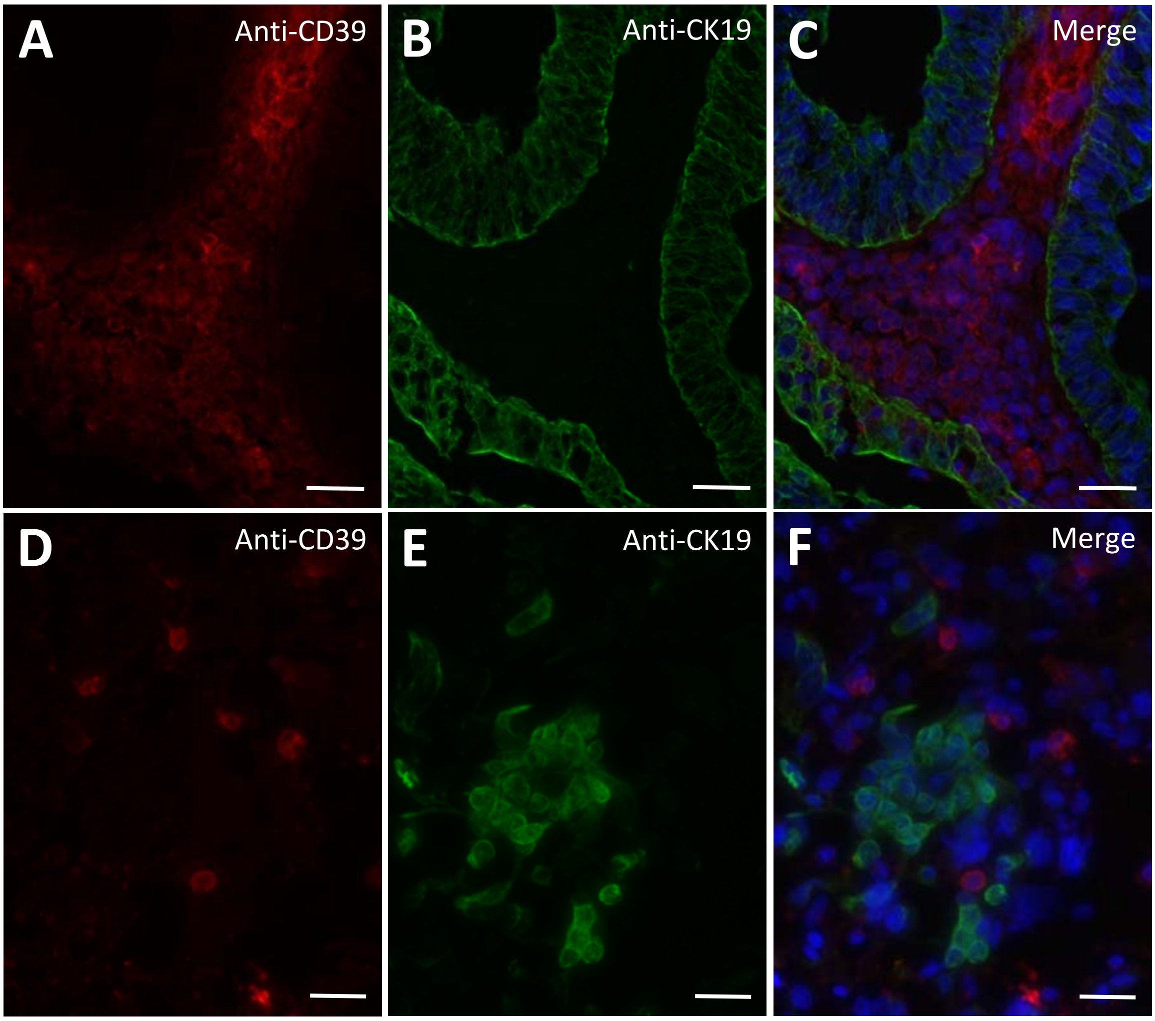

Supplementary figure 2

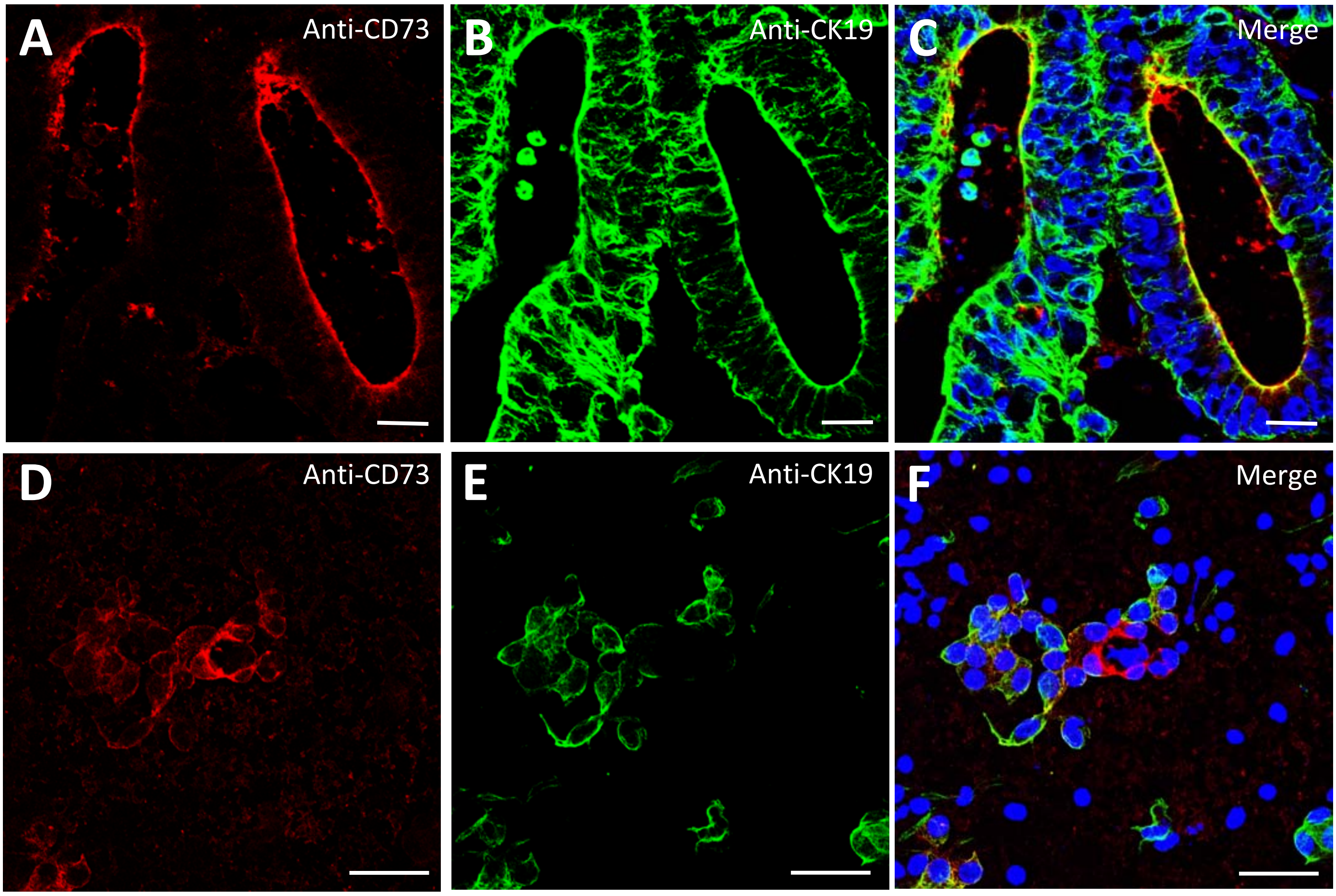

Supplement: Supplementary file 1 — The supplementary figures demonstrate that CD39 does not colocalize with CK19 (Figure 1) whereas CD73 partially colocalizes (Figure 2). [file 509027.f1.pdf]
